# A multi-omic assessment of the mechanisms of intestinal microbes used to treat diarrhea in early-weaned lambs

**DOI:** 10.1128/msystems.00953-23

**Published:** 2024-01-09

**Authors:** Hongran Guo, Jiuzeng Cui, Qian Li, Xuhui Liang, Junda Li, Bohua Yang, Peter Kalds, Yulin Chen, Yuxin Yang

**Affiliations:** 1 Key Laboratory of Animal Genetics, Breeding and Reproduction of Shaanxi Province, College of Animal Science and Technology, Northwest A&F University, Yangling, Shaanxi Province, China; Duke University School of Medicine, Durham, North Carolina, USA

**Keywords:** lambs, early-weaning stress, diarrhea, fecal microbiota transplantation

## Abstract

Transplant of donor microbiota can significantly alter the structure of the host’s intestinal microbiota and alleviate early weaning stress. Screening for alternative-resistant products by transplanting fecal bacteria from healthy lambs is a current research trend in the livestock industry. In the present study, fecal microbiota transplantation was performed in lambs with diarrhea during early weaning. The transplanted fecal microbiota greatly reduced the diarrhea and serum inflammatory factor levels caused by early weaning. Transcriptome sequencing revealed that fecal microbiota transplantation alleviated colonic inflammation and increased the expression of colonic ion transport proteins. In addition, the levels of *Streptococcus*, *Enterococcus,* and *Escherichia Shigella* decreased in the jejunum, cecum, and colon of the lambs; meanwhile, the levels of *Bifidobacterium* and multiple secondary bile acids, such as ursodeoxycholic acid, increased in the colon. Furthermore, the abundance of *Bifidobacterium* was significantly negatively correlated with the diarrhea index. The fecal microbiota transplantation reshaped the intestinal microbiota of early-weaned lambs, protected the intestinal physiology and immune barrier, and reduced weaning stress. In addition to making available bacteriological products for controlling intestinal inflammation in young lambs, this study offers a theoretical framework and technical system for the mechanisms by which microbiota transplantation regulates intestinal health in young lambs.

**IMPORTANCE:**

Before weaning, the digestive system of lambs is not well developed; hence, its resistance to infectious diseases is weak. Under intensive feeding systems, lambs can easily be stressed and the risk of bacterial infection is high, which causes diarrhea, which in turn may cause mortality and significant economic losses to the livestock industry. With the elimination of antibiotics in animal feed, the incidence of mortality due to intestinal illnesses in lambs has gradually increased. There are several types of probiotics routinely used in young animals, but the effects and processes of their usage have only been assessed in monogastric animals. The lack of data on ruminants, particularly sheep, has severely hampered the process of efficient and healthy sheep breeding. Therefore, there is an urgent need to identify effective and safe functional supplements for lambs.

## INTRODUCTION

Early weaning accelerates sheep reproduction and reduces the length of the breeding cycle. As the immune systems of lambs are developing, early weaning stress has a considerable negative impact on growth performance and intestinal health (1). Early weaning stress in lambs can consequently result in diarrhea and mortality. Antibiotics are currently the most common treatment strategy to manage pathogenic diseases in domestic animals; however, their use is associated with the development of resistant bacteria and antibiotic residues in food-producing animals (2). Consequently, one of the most popular areas of research has been the development of functional microbial-based formulations to replace antibiotics (3).

Sheep intestines contain tens of thousands of bacteria that interact with the host to maintain intestinal homeostasis (4). Weaning stress disturbs the intestinal microbiota in lambs, thereby impairing intestinal functions. Fecal microbiota transplantation (FMT) technology is used to regulate intestinal bacteria and metabolites in young animals; it facilitates the transfer of fecal microbiota from healthy donors to infected recipients. In weaning piglets, FMT helps to restore the balance of intestinal flora and relieve intestinal damage caused by weaning stress (5).The investigatory method for FMT in piglets has been well developed; however, the effects and mechanism of FMT in sheep remain unknown. This is due in part to the unique gastrointestinal tract structure in sheep.

The aim of our study is to apply FMT-based screening on probiotics that improve intestinal microbiota homeostasis and reduce intestinal inflammation and diarrhea in lambs caused by early weaning. Herein, lambs were subjected to early weaning and fed milk formula. The early-weaned lambs were treated with FMT, and their phenotype was observed. We hypothesize that FMT in lambs can reduce the symptoms of diarrhea by altering the intestinal microbiota composition and boosting intestinal beneficial bacteria and metabolite abundance. Such an increased abundance of beneficial intestinal bacteria can mitigate intestinal inflammation.

## MATERIALS AND METHODS

### Preparation of fecal supernatants from donor sheep for transplantation

Six healthy sheep (three male and three female) aged 60–90 d were selected as fecal bacterial donors from a sheep farm located in the Gansu province, China. The donor sheep had never experienced diarrhea, consumed probiotics or antibiotics, or previously been exposed to any infectious diseases. Each donor sheep provided 6.5 g of feces, which were combined at a 1:10 ratio with phosphate-buffered saline (PBS). After being filtered three times using a sterile 0.25 mm sieve, the mixture was centrifuged at 800 × *g* for 2 min at 4°C. Each sheep’s fecal supernatant was collected in equal amounts and stored in liquid nitrogen containing 30% glycerol. The stored supernatants were thawed in a 37°C water bath for 1 h prior to transplantation. The *in vitro* culture was prepared and counting of viable bacteria was done to ensure that the viable count was >10^9^ CFU/mL per tube (6).

### Lamb farming and sample collection

Forty lambs from the same flock, with a similar age (3 days old) and weight, were selected after birth (Fig. S1). At 10 days of age, the lambs stopped nursing and were fed a milk replacer (Lamb Formula, Nutrifeed) (Table S1). The lambs experienced watery diarrhea at 14 days, and 18 lambs were randomly separated into three groups (*n* = 6 per group). The PBS used in the negative control group (PBS group) was infused at a volume of 4 mL each day. The positive control group (antibiotics [ABX] group) received daily antibiotic injections (lambs were orally administered 10 mg of gentamycin sulfate per kilogram of body weight and 10,000 units of penicillin per kilogram of body weight by injection). Each sheep in the experimental group (FMT group) was administered 4 mL of the fecal supernatant intragastrically. Six lambs without the diarrhea phenotype were used as controls (health [HEA] group). Lamb weight and diarrhea status were recorded every 3 days during the 14-day testing period. The lamb diarrhea index was calculated as shown in Table S2. Blood samples were collected at the end of the testing period. After the lambs were slaughtered, the contents of the jejunum, cecum, and colon were collected, and the colonic tissues were kept in a freezer at −80°C. Colonic tissue was collected and fixed in 4% paraformaldehyde.

### Serum index testing

The collected blood samples were centrifuged at 1,789 *× g* for 10 min. Serum was extracted and stored at −80℃ for subsequent testing using ELISA kits to detect the levels of interleukin-6 (IL-6), tumor necrosis factor alpha (TNF-α), and IL-1β (FANKEW, Shanghai, China).

### RNA extraction and RNA-seq data analysis

Total RNA was extracted from the colon tissues using the Trizon reagent (TaKaRa, Dalian, China), according to the manufacturer’s instructions. RNA quantification was performed using a Nanodrop2000 ultraviolet (UV)-vis spectrophotometer (Thermo Scientific, Wilmington, USA) to detect purity and concentration.

Agarose gel electrophoresis was conducted to determine the RNA integrity. The 2100 Bioanalyser (Agilent) was used to determine the RNA integrity number (RIN) value (RIN >8.0) (7). The extracted RNA was used to construct a library using the Truseq TM RNA sample prep kit (Illumina, San Diego, CA). Total library concentration was assayed using TBS380, and the constructed libraries were sequenced using Illumina HiSeq 4000 (2 × 150 bp read length). SeqPrep and Sickle with default parameters were applied to trim and control raw paired-end reads (1). Clean reads were compared with the sheep reference genome (Oar_rambouillet_v1.0) via TopHat (v2.1.1). Fragments per kilobase million (FPKM) and transcripts per kilobase million (TPM) values were calculated to normalize the expression (RSEM v1.3.3). Differential analysis of gene expression was performed using DESeq2 software (v1.24.0).

### Real-time PCR analysis

Total RNA was extracted from the colon tissues using the Trizon reagent (TaKaRa, Dalian, China), according to the manufacturer’s instructions. RNA was reverse transcribed to cDNA using the HiScript II Q RT SuperMix for qPCR Kit (R223-01, Vazyme, Nanjing, China), following the manufacturer’s instructions. The real-time fluorescence quantitative PCR (qPCR) reaction system was constructed using ChamQ SYBR qPCR Master Mix (Q311-02, Vazyme, Nanjing, China). The qPCR system was as follows: 0.4 µL of each of the forward and reverse primers, 10 µL of 2 × ChamQ SYBR qPCR Master Mix, 2 µL of cDNA template, and 7.2 µL of water. The reaction conditions were set as follows: 95℃ for 30 s, 95℃ for 10 s, and 60℃ for 30 s, with a total of 40 cycles, while the melting reaction conditions were as follows: 95℃ for 15 s, 60℃ for 60 s, and 95℃ for 15 s. Real-time fluorescence quantification was used to determine the relative expression of the *DRA*, *PAT1*, *NHE3*, and *SLC5A1* genes in the intestinal tissues. The primer sequences used in qPCR are listed in Table S3.

### H&E staining

Colonic tissues from sheep were fixed with 4% (vol/vol) paraformaldehyde overnight at 4°C, dehydrated with ethanol, and embedded in liquid paraffin. Paraffin sections of 5–8 mm were prepared from the paraffin-embedded tissue. Xylene ethanol dewaxed and hydrated the paraffin sections, and the dewaxed sections were stained with hematoxylin-eosin (H&E) solution (8).

### 16S rRNA gene sequencing

The Stool DNA kit D4015 (OMEGA, America) was used to extract DNA, and 1% agarose gel electrophoresis was used to identify the DNA in the jejunum, cecum, and colon contents. The target portion of the bacterial 16S rRNA gene’s V3-V4 region was amplified using universal bacterial primers 338F and 806R (Table S3) (1). Pairwise sequencing of equimolar ratios of purified amplicons was performed on the Illumina MiSeq platform according to the standard protocol of Major Biobio-Pharm Technology Co. Ltd. (Shanghai, China). The raw sequencing data were spliced for quality control and sequence noise reduction using FLASH (v1.2.7), fastp (v0.19.6), and DADA2. To obtain the taxonomic information of the species corresponding to each amplicon sequence variant (ASV), the representative sequences of ASVs were taxonomically analyzed using the classify-sklearn method (Naive Bayes; Qiime2 v2022.2), and the annotated information of the ASVs at the taxonomic level was obtained by comparing the databases silva138/16 s_bacteria (confidence level >0.7). The alpha diversity of the gut microbiota was analyzed using mothur-1.30, while the beta diversity of the gut microbiota was analyzed using R-3.3.1 (vegan).

### Metabolomics

Sheep’s colonic contents were homogenized using a 500 mL solution of cold methanol and water (70%, vol/vol). Following a 10 min ultrasonic treatment in an ice-water bath, samples were swirled for 3 min and centrifuged for 10 min at 4°C (16,099 × *g*). The liquid chromatography-tandem mass spectrometry (LC-MS/MS) analysis (UPLC, Shim-pack UFLC SHIMADZU CBM A system, https://www.shimadzu.com/; MS, QTRAP system, https://sciex.com/) was then performed using the obtained supernatants (9).

### Data analysis

|Log2 (fold change)| > 0 and *P* < 0.05 were required for the RNA-seq screening for differentially expressed genes (DEGs). *P* < 0.05 for the gene ontology (GO) enrichment analysis was considered significant. Microorganisms were analyzed for between-group differences using the Kruskal-Wallis H test with a significance level of 0.05, multiple test correction of fdr, and post-hoc Tukey-Kramer test of 0.95. All data are expressed as the mean ± standard error (SEM) using GraphPad Prism (v8.3, GraphPad, USA). Statistical analyses were performed using SPSS software (v19.0, IBM, USA). Statistical differences were defined as *P < 0.05, **P < 0.01, and ***P < 0.001.

## RESULTS

### FMT relieves milk replacer-induced diarrhea in lambs

**Fig 1:**
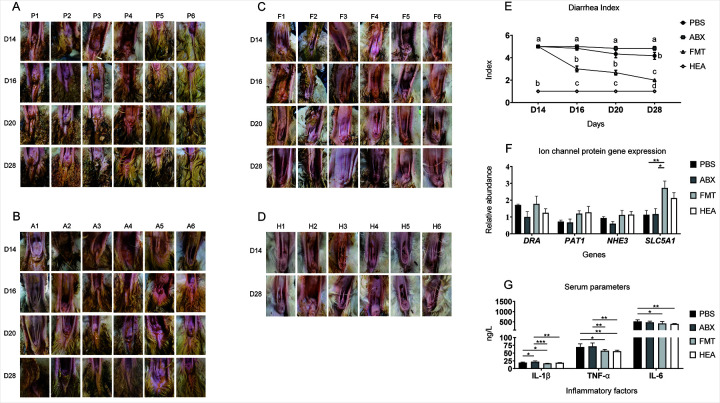
FMT alleviates stress in early-weaned lambs. Diarrhea in lambs in the (**A**) PBS, (**B**) ABX, (**C**) FMT, and (**D**) HEA groups. (**E**) Changes in the diarrhea index during the testing period. (**F**) Ion channel protein expression in the colon tissue. (**G**) Serum levels of inflammatory factors. Mean ± SEM is shown (*n* = 6). *P < 0.05, **P < 0.01, and ***P < 0.001. The letters (a, b, c, and d) in panel E represent the two sets of significant differences at different time periods.

To determine whether gut microbes resist nutritional diarrhea, we prepared manure from healthy donor lambs and intragastrically administered it to recipient lambs with diarrhea. The results showed that the microbiota transplanted from the healthy lambs, but not from the PBS or mixed antibiotics, significantly alleviated diarrhea symptoms caused by the early weaning stress (*P* < 0.01; Fig. 1A through E). Additionally, there was a significant increase in the expression of fluid absorption (*SLC5A1*) in the FMT group (*P* < 0.01; Fig. 1F).

### Serum parameters

Serum IL-1β levels were significantly higher in the ABX than PBS groups but significantly lower in the FMT and HEA groups (*P* < 0.01). Serum TNF-α (*P* < 0.01) and IL-6 levels (*P* = 0.02) were also considerably reduced in the FMT and HEA groups when compared with those in the PBS group and did not change significantly in the ABX group (Fig. 1G). These results demonstrate that the transplantation of healthy fecal bacteria into diarrheic lambs negatively affects inflammatory markers.

### FMT alleviates colonic inflammation in diarrheic lambs

**Fig 2:**
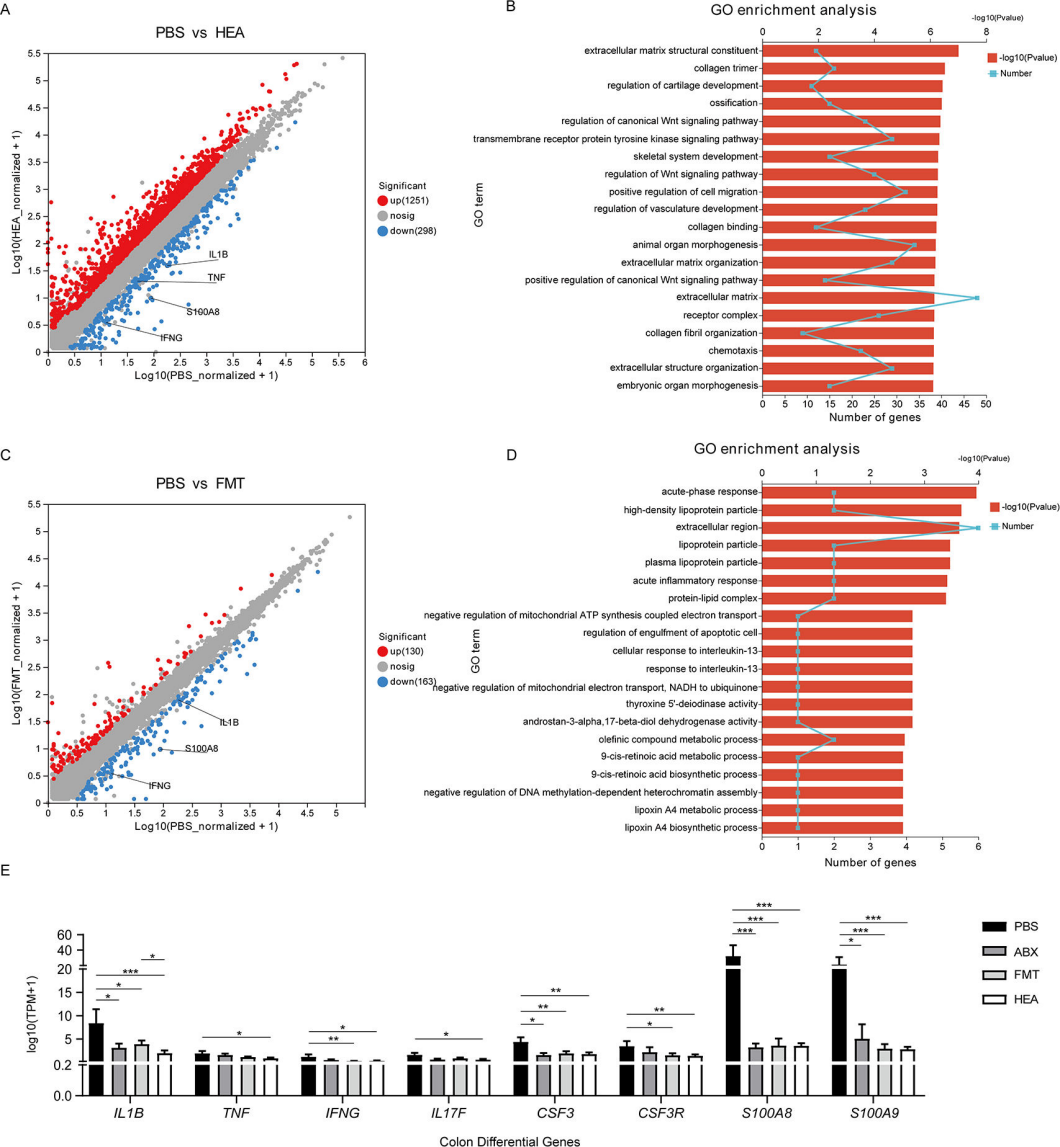
FMT alters lamb colonic gene expression. Volcano map (**A**) and Kyoto Encylopedia of Genes and Genomes (KEGG) enrichment analysis (**B**) of the differential genes in the PBS vs HEA groups. Volcano map (**C**) and KEGG enrichment analysis (**D**) of the differential genes in the PBS vs FMT groups. (**E**) Changes in gene expression related to colonic inflammation. Mean ± SEM is shown (*n* = 4). *P < 0.05, **P < 0.01, and ***P < 0.001.

RNA-seq was used to analyze the effects of transplanted fecal bacteria on colonic gene expression. A total of 170.05 Gb of clean data were obtained from the transcriptome analysis of the 24 samples, and there were >6.07 Gb clean data from the sample, with a percentage of Q30 bases >93.33%. After quantitative analysis of the genes sequenced and their read counts, differential expression analysis of the genes between the groups was performed on multiple samples. Compared with the PBS group, there were 1,656 upregulated and 446 downregulated genes in the HEA group (Fig. 2A), and 149 upregulated and 210 downregulated genes in the FMT group (Fig. 2C).

The obtained differential genes were subjected to GO enrichment analysis, and the top 20 significant enriched signaling pathways were identified. DEGs in the HEA group were enriched in chemotaxis, and acute inflammatory responses were compared with the PBS and FMT groups (Fig. 2B and D). In contrast to the PBS group, the expression of inflammation-related genes (*IL1B*, *TNF*, *IFNG*, *S100A8*, *S100A9*, *CSF3*, and *CSF3R*) significantly decreased in the ABX, FMT, and HEA groups (*P* < 0.02). In addition, there was a significant reduction in colonic inflammation in the FMT and HEA groups (Fig. S3).

### FMT shifts the gut microbiota structure in diarrheic lambs

**Fig 3:**
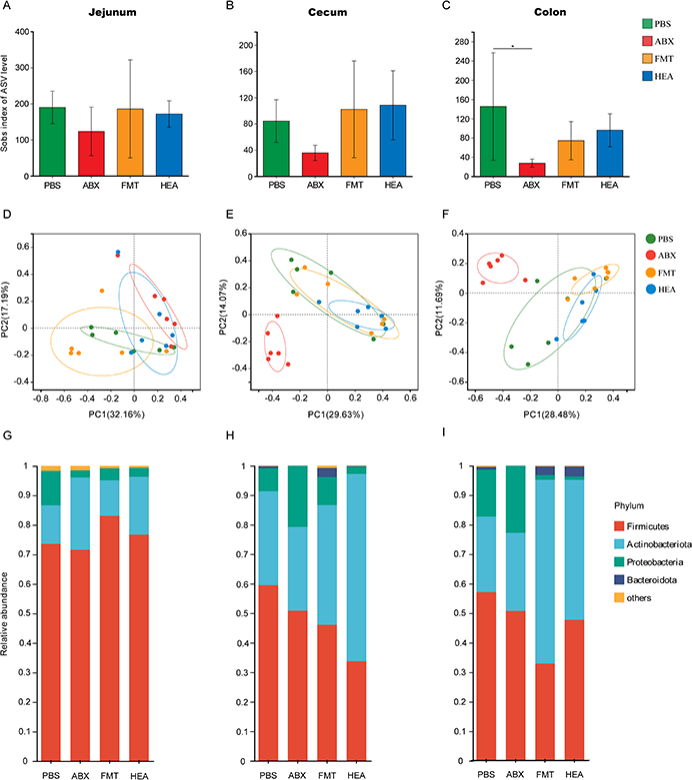
FMT alters the structure of the intestinal microbiota. Alpha diversity of the jejunum (**A**), cecum (**B**), and colon (**C**) contents. Beta diversity of the jejunum (**D**), cecum (**E**), and colon (**F**) contents. Phylum level abundance of the jejunum (**G**), cecum (**H**), and colon (**I**) contents. *P < 0.05.

To observe the diversity of the gut microbiota structure of lambs, the contents of the jejunum, cecum, and colon were obtained using 16S rRNA gene sequencing. The Chao index in the colon significantly decreased in the ABX group compared with the PBS group (*P* < 0.01), and there were no significant changes in the jejunum (*P* = 0.32) (Fig. 3A through C). According to principal coordinate analysis (PCoA), the jejunum microbes in the ABX group were significantly separated from those in the PBS and FMT groups. In the cecum and colon, the separation was significant in the ABX group from the three other groups (Fig. 3D through F).

FMT also altered the abundances of the most prevalent bacterial phyla, including *Firmicutes*, *Actinobacteriota*, *Proteobacteria*, and *Bacteroidota* (Fig. 3G through I). In the jejunum, the abundance did not differ significantly. The abundance of *Bacteroidota* changed significantly in the cecum as the intestinal contents moved backward (*P* = 0.005). In the colon, the abundance of most phyla showed clear variation. Indeed, the abundance of *Actinobacteriota* in the FMT group was significantly higher than that in the PBS group (*P* = 0.03), while the abundance of *Proteobacteria* (*P* = 0.004) and *Bacteroidota* (*P* = 0.007) changed significantly (Fig. S4).

### FMT alters the abundance of intestinal bacteria

**Fig 4:**
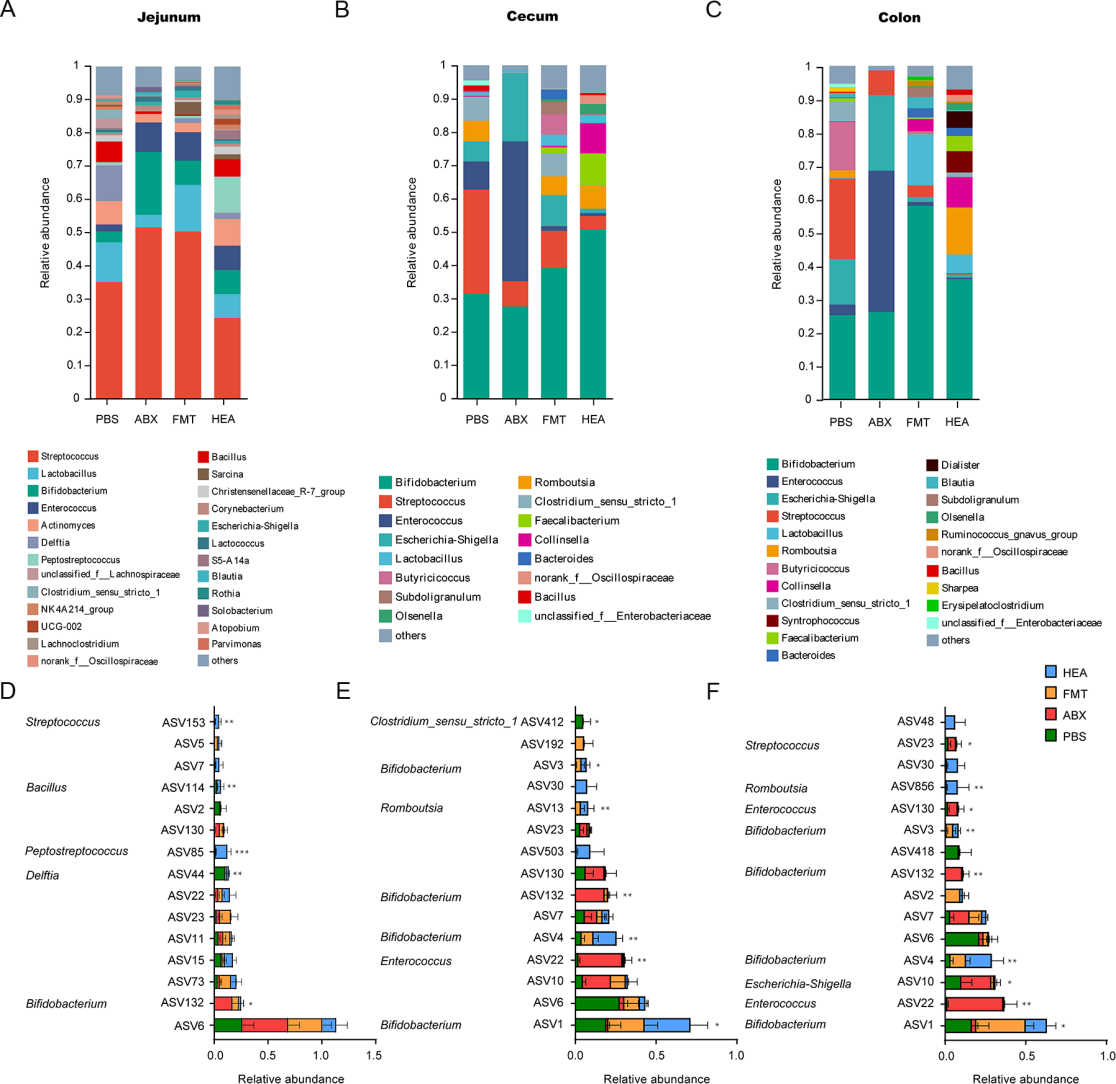
FMT alters the abundance of intestinal bacteria. Genus-level abundance of jejunum (**A**), cecum (**B**), and colon (**C**) contents. ASV abundance in the jejunum (**D**), cecum (**E**), and colon (**F**) contents. ASVs are listed with the corresponding genus. *P < 0.05, **P < 0.01, and ***P < 0.001.

To determine whether specific bacteria were resistant to diarrhea, we compared the changes in gut bacteria. In the jejunum, at the genus level, the abundance of *Delftia* was significantly higher in the PBS group (*P* < 0.01), while the abundances of *Peptostreptococcus* (*P* < 0.01), *Lachnoclostridium* (*P* = 0.02), and *norank_f__Oscillospiraceae* (*P* < 0.01) were significantly higher in the HEA group compared with the other groups. The abundances of *Bacillus* (*P* = 0.01), *Christensenellaceae_R-7* (*P* < 0.01), *Parvimonas* (*P* < 0.01), and *Solobacterium* (*P* = 0.03) varied significantly between groups (Fig. 4A). In the cecum, the abundance of *Enterococcus* was higher in the ABX group compared with the other groups (*P* < 0.01), while that of *Romboutsia* (*P* = 0.01), *Bacteroides* (*P* = 0.04), *norank_f__Oscillospiraceae* (*P* = 0.01), and *Bacillus* (*P* < 0.01) changed significantly between the four groups (Fig. 4B).

In the colon, the abundance of *Escherichia-Shigella* increased in the ABX group compared with the other groups (*P* = 0.01), while that of *Enterococcus* (*P* < 0.01), *Romboutsia* (*P* < 0.01), *Collinsella* (*P* = 0.04), *Bacteroides* (*P* = 0.04), *Dialister* (*P* < 0.01), and *Bacillus* (*P* < 0.01) were altered between the four groups (Fig. 4C).

The common ASVs were then assessed in the jejunum, cecum, and colon. In the jejunum, the abundance of *Peptostreptococcus* (ASV85) was markedly higher in the HEA group compared with the other groups (*P* < 0.01), while the abundance of *Delftia* (ASV44) was significantly elevated in the PBS group (*P* < 0.01) (Fig. 4D). In the cecum, the abundances of *Enterococcus* (ASV22) (*P* < 0.01) and *Bifidobacterium* (ASV132) (*P* < 0.01) increased significantly in the ABX group, while that of *Bifidobacterium* (ASV4) was significantly increased in the HEA group (*P* < 0.01). The abundance of *Bifidobacterium* (ASV1 [*P* = 0.04] and ASV3 [*P* = 0.01]) was significantly different between the groups (Fig. 4E). In the colon, the abundances of *Enterococcus* (ASV22) (*P* < 0.01), *Escherichia-Shigella* (ASV10; *P* = 0.01), and *Bifidobacterium* (ASV132; *P* < 0.01) increased significantly in the ABX group. However, the abundance of *Bifidobacterium* (ASV1 [*P* = 0.01] and ASV3 [*P* < 0.01]) significantly increased in the FMT group compared with the ABX group. *Bifidobacterium* (ASV4) was significantly increased in the HEA group (*P* < 0.01; Fig. 4F).

### Gut microbes are associated with diarrhea and serum inflammatory markers

To correlate the gut microbiota with the diarrhea and serum inflammatory markers, Spearman’s correlation analysis was performed using the significantly altered ASVs with the diarrhea index and serum inflammatory factors in lambs. The thermogram showed a correlation between the phenotype and ASVs in the jejunum, cecum, and colon (Fig. S5). ASVs belonging to the genera *Bifidobacterium* (ASV1, ASV3, and ASV4) and *Collinsella* (ASV30) were negatively correlated with the diarrhea index (*P* < 0.01).

The abundance of *Bifidobacterium* (ASV1, ASV3, and ASV4) in the cecum and colon had an adverse correlation with IL-1β and TNF-α (*P* < 0.01). In contrast, ASVs belonging to the genera *Streptococcus* (ASV23), *Enterococcus* (ASV130 and ASV22), and *Escherichia-Shigella* (ASV10) were significantly positively correlated with the diarrhea index (*P* < 0.01).

### Intestinal microbiota associated with bile acid metabolism

**Fig 5:**
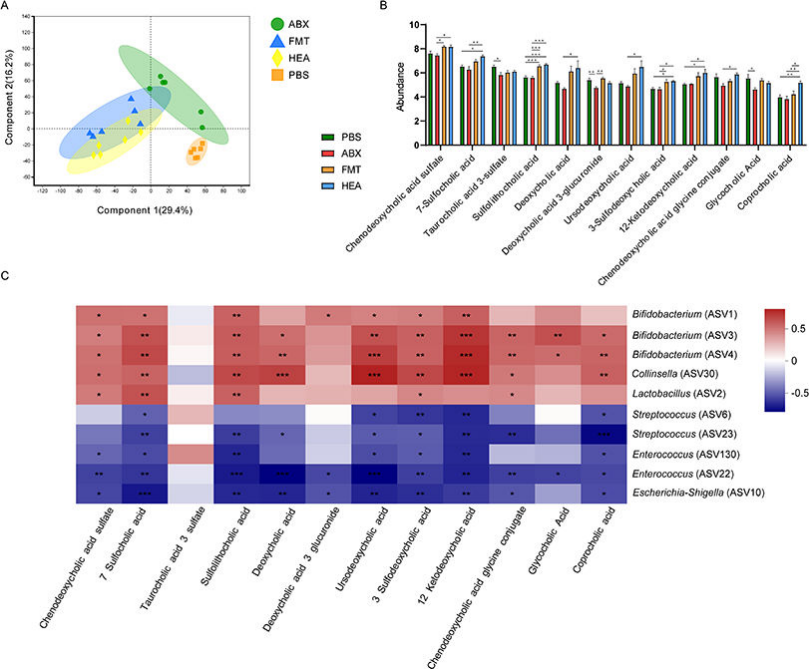
FMT varies the levels of colon metabolites. (**A**) PLS-DA analysis. (**B**) Impact on bile acid levels and effects on bile acid-related metabolites. (**C**) Correlation analysis of bile acid-related metabolites with significantly different bacteria at the genus level. Red indicates a positive correlation, and blue indicates a negative correlation. *P < 0.05, **P < 0.01, and ***P < 0.001.

Untargeted metabolomics were used to determine the small-molecule metabolite levels in the colon. A total of 1,050 cations and 730 anions were identified in the intestine. According to the partial least squares discriminant analysis (PLS-DA) analysis, the FMT and HEA groups clearly differed from the PBS group (Fig. 5A). The Human Metabolome Database (HMDB) compound classification results showed that the metabolites could be classified into 12 classes, according to the positive and negative patterns, of which 48.57% were annotated as lipids or lipid-like molecules (Fig. S6). At the same time, significant differences were found in the bile acid levels between groups (Fig. 5B). The abundance of chenodeoxycholic acid sulfate (*P* < 0.01), sulfolithocholic acid (*P* < 0.01), and 3-sulfodeoxycholic acid (*P* < 0.01) increased significantly in the FMT and HEA groups. Abundant 7-sulfocholic acid (*P* < 0.01), deoxycholic acid (*P* < 0.01), ursodeoxycholic acid (*P* < 0.01), 12-ketodeoxycholic acid (*P* < 0.01), chenodeoxycholic acid glycine conjugate (*P* < 0.01), and coprocholic acid (*P* < 0.01) were observed in the HEA group. In addition, the abundance of taurocholic acid 3-sulfate (*P* = 0.04) and glycolic acid (*P* < 0.01) was significantly higher in the PBS than ABX groups.

Correlation analysis was used to correlate variable ASVs with variable bile acids, which showed that all metabolites, except for taurocholic acid 3-sulfate and deoxycholic acid 3-glucuronide, were significantly positively correlated with *Bifidobacterium* (ASV3 and ASV4; *P* < 0.01). The abundance of chenodeoxycholic acid sulfate, 7-sulfocholic acid, and sulfolithocholic acid was significantly positively correlated with *Bifidobacterium* (ASV1, ASV3, and ASV4), *Collinsella* (ASV30), and *Lactobacillus* (ASV2) (*P* < 0.05). In contrast, *Streptococcus* (ASV6 and ASV23), *Enterococcus* (ASV130 and ASV22), and *Escherichia-Shigella* (ASV10) were significantly negatively correlated with the metabolites (*P* < 0.05) (Fig. 5C). Megablasts were also used to compare ASV3 and ASV4 and the comparison results for *Bifidobacterium breve* and *Bifidobacterium bifidum*, respectively.

## DISCUSSION

There is an urgent need to explore functional probiotics to replace antibiotics in animal breeding (10). The results of this study showed that FMT and transplanted fecal bacteria could significantly alleviate early weaning-induced diarrhea and intestinal inflammation in lambs and increase the abundance of beneficial bacteria in the intestine. These results indicated that FMT could replace antibiotics to prevent early weaning stress. Furthermore, increased abundance of *Bifidobacterium* and *Collinsella* was correlated with increased secondary bile acids in the intestine. The enrichment of intestinal probiotics was significantly associated with the relief of inflammation in lambs. This study provides data that support the application of FMT technology in ruminants, along with a theoretical basis and technical system to elucidate the mechanisms by which *Bifidobacterium* regulates the intestinal health of young lambs.

Early weaning increases intestinal inflammation (11), triggering diarrhea in lambs. The present study demonstrated FMT as a potential treatment for diarrhea caused by early weaning. Although lambs have a complex stomach structure, transplanted bacteria can still reach the hindgut and exert anti-diarrheal effects (12). The pathogenesis of diarrhea involves the excessive secretion of CL^-^ in the intestine and inhibition of Na^+^ absorption. The membrane proteins involved in Na^+^ uptake include SLC5A1 (sodium-glucose cotransporter) and NHE3 (Na^+^ uptake and H^+^ exclusion) (13). In this study, FMT significantly increased the absorption and steady-state (*SLC8A3*, sodium-calcium exchanger) of Na^+^ in the colon. In addition, intestinal inflammation in the host can trigger diarrhea. Early weaning stress triggers the expression of pro-inflammatory genes (*IL1B*, *TNF*, *IFNG*, and *IL17F*) by intestinal tissue cells. Gamma interferon (IFN-γ) and IL-17F promote macrophage activation (*TNF*, *S100A8*, *S100A9*, and *CSF3*) and aggregation in intestinal tissues, exacerbating the inflammatory process. FMT significantly reduces the expression of these inflammatory genes and decreases the inflammatory response in lambs (14).

FMT has been reported to restore antibiotic-induced microbiota disorders in mice and inhibit the enrichment of commensal pathogenic bacteria such as *Klebsiella* and *E. shigella* (15). FMT treats colitis by improving the structure and function of the patient’s microbiota (16). Patients with immune-mediated colitis who underwent fecal transplantation for symptomatic relief showed elevated alpha diversity of the intestinal bacteria and *Collinsella* and *Bifidobacterium* (17); the same results were obtained in this study. *Collinsella* is a signature genus used to distinguish healthy calves from enteroaggregative *Escherichia coli*-infected calves with diarrhea and may be associated with an increased abundance of secondary bile acids (18). *Bifidobacterium* dominate the intestinal tract during the early stages of life (19) and help to regulate immunity (20). Increasing the abundance of *Bifidobacterium* significantly reduces the relative abundance of commensal pathogenic bacteria (21). Moreover, short-term supplementation of *Bifidobacterium* to breastfed infants reduces the abundance of potentially pathogenic bacteria and virulence factor genes (22). Additionally, *Bifidobacterium* is interfacially active and acts as a primary degrader of complex carbohydrates, producing metabolites that can be used as fermentation substrates by other bacteria (23). Besides, *Lactobacillus* synergistically optimizes biological, chemical, and mechanical barriers through activation of relevant metabolic pathways, which in turn modulates inflammation (24).

Secondary bile acids in the intestine depend on the conversion of intestinal bacteria into bile acids (25). Decreased levels of lithobiliary acid (LCA) and deoxycholic acid (DCA) in the intestine inhibit the TGR5 pathway and promote intestinal inflammation (26). In addition, DCA has potent antibacterial activity and is effective for the prevention and treatment of bacterial infections (27). Chenodeoxycholic acid (CDCA) effectively inhibits the expression of virulence genes and epithelial cell invasion by *Salmonella typhimurium* (28). Ursodeoxycholic acid (UDCA) reduces the production of inflammatory cytokines by binding to the bile acid receptor farnesoid X receptor (FXR) and inhibits NF-κB activation in macrophages (29). UDCA inhibits pathogenic bacterial growth and invasion both *in vitro* and *in vivo* via the TGR5-NFκB signaling axis, attenuates commensal bacterial dysbiosis during extended-spectrum β-lactamase-producing enteroaggregative *Escherichia coli* (ESBL-EAEC) infection, and upregulates hindgut short chain fatty acid (SCFA) levels against calf diarrhea in neonatal mouse models for sepsis and colitis (18). In this investigation, FMT significantly increased secondary bile acid levels in the colon, which might have played a role in relieving weaning stress. However, whether there is a causal relationship between beneficial bacteria and secondary bile acids remains to be determined; only a positive correlation was observed in this study. Further investigations are warranted to investigate how beneficial bacteria increase secondary bile acid concentrations in the intestine and the effects of secondary bile acids on the alleviation of early weaning stress in lambs.

### Conclusions

This study showed that FMT alleviated early weaning-induced diarrhea in lambs by increasing the abundance of beneficial bacteria in the intestine and secondary bile acid content and decreasing the level of intestinal inflammation. The results provide a crucial theoretical underpinning and technical framework for the potential use of prebiotics as regulators of intestinal health in young lambs.

## Data Availability

All data generated or analyzed during this study are included in this published article and its supplemental information files. Sequencing data were uploaded to the National Center for Biotechnology Information (NCBI) database: PRJNA956277 and PRJNA951809.

## AFTER EPUB

*[This article was published on 9 January 2024 with information missing from the Author Contributions and some references. The Author Contributions and References were corrected in the current version, posted on 23 January 2024.]*

## SUPPLEMENTAL MATERIAL

Figure S1 msystems.00953-23-s0001.tif Experimental design and procedure of the lamb test.

Figure S2 msystems.00953-23-s0002.tif The weights of lambs recorded during the experiment.

Figure S3 msystems.00953-23-s0003.tif Hematoxylin-eosin staining of colonic tissue in the lambs.

Figure S4 msystems.00953-23-s0004.tif FMT significantly alters the abundance of intestinal bacteria. phylum level abundance of cecum (A) and colon (B) contents. *, *P* < 0.05.

Figure S5 msystems.00953-23-s0005.tif Correlation analysis of intestinal bacteria with diarrhea index and serum inflammatory factor levels in lambs. Red represents a positive correlation, while blue represents a negative correlation. JE, jejunum; CE, cecum; CO, colon. *, *P* < 0.05; **, *P* < 0.01; ***, *P* < 0.001.

Figure S6 msystems.00953-23-s0006.tif FMT altered the metabolites of the colonic contents of weaned lambs. Taxonomic information and enrichment analysis of metabolites in colonic contents. (A) The detected metabolites were classified as HMDB compounds. (B) The differential metabolites of the four groups were subjected to KEGG pathway enrichment analysis.

Table S1 msystems.00953-23-s0007.xlsx Typical composition of milk replacer.

Table S2 msystems.00953-23-s0008.xlsx Criteria for diarrhea index scoring in lambs.

Table S3 msystems.00953-23-s0009.xlsx The quantitative primer sequences.

Table S4 msystems.00953-23-s0010.csv Content of metabolites in colonic contents.

ASM does not own the copyrights to Supplemental Material that may be linked to, or accessed through, an article. The authors have granted ASM a non-exclusive, world-wide license to publish the Supplemental Material files. Please contact the corresponding author directly for reuse.
