# Supplementary figures and images for: A multi-omic assessment of the mechanisms of intestinal microbes used to treat diarrhea in early-weaned lambs
Source: mSystems. 2024 Jan 9;9(2):e00953-23. doi: 10.1128/msystems.00953-23 (PMC10878098; doi:10.1128/msystems.00953-23)

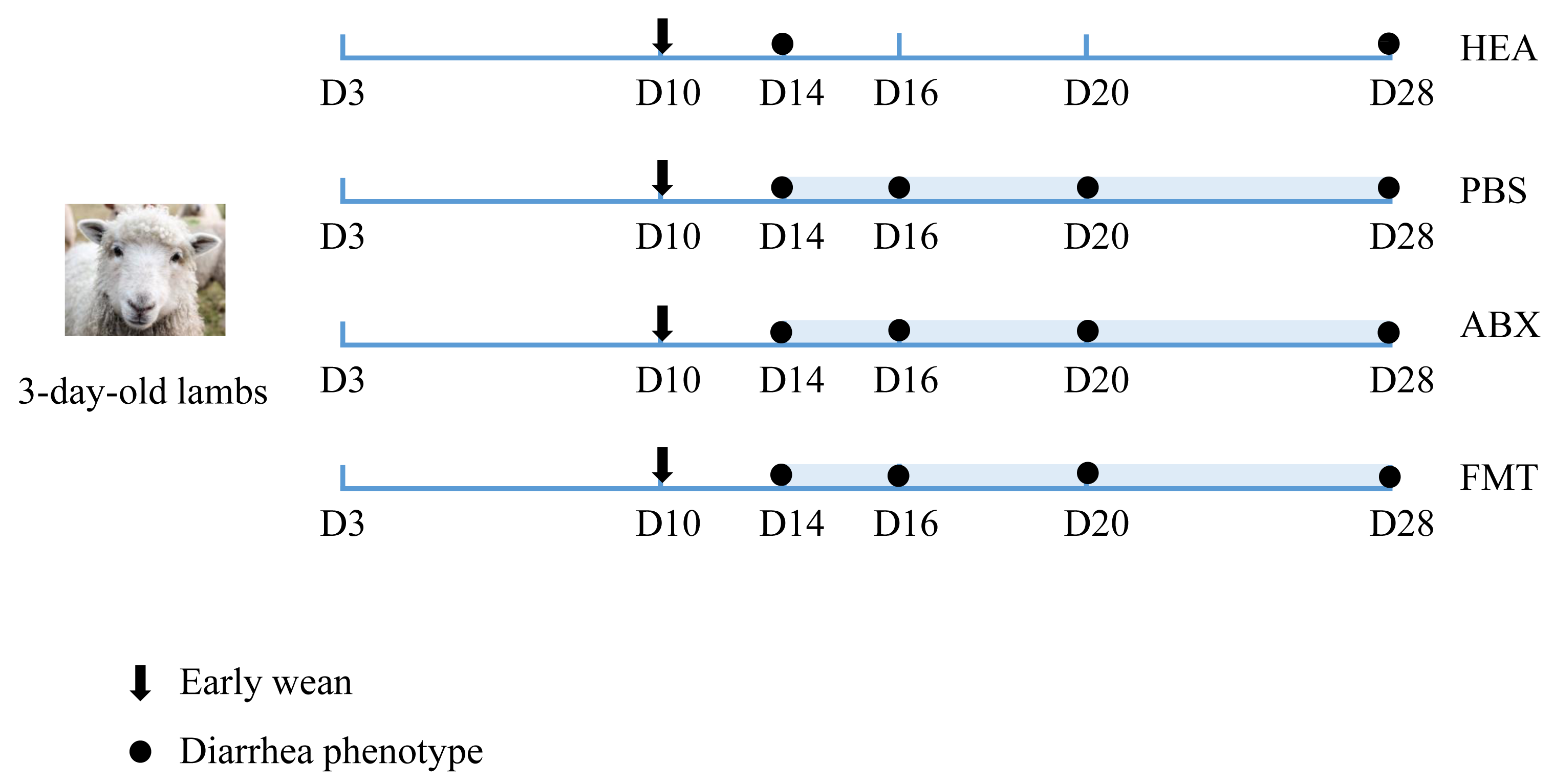

Supplement: Figure S1 — Experimental design and procedure of the lamb test. [file msystems.00953-23-s0001.tif]

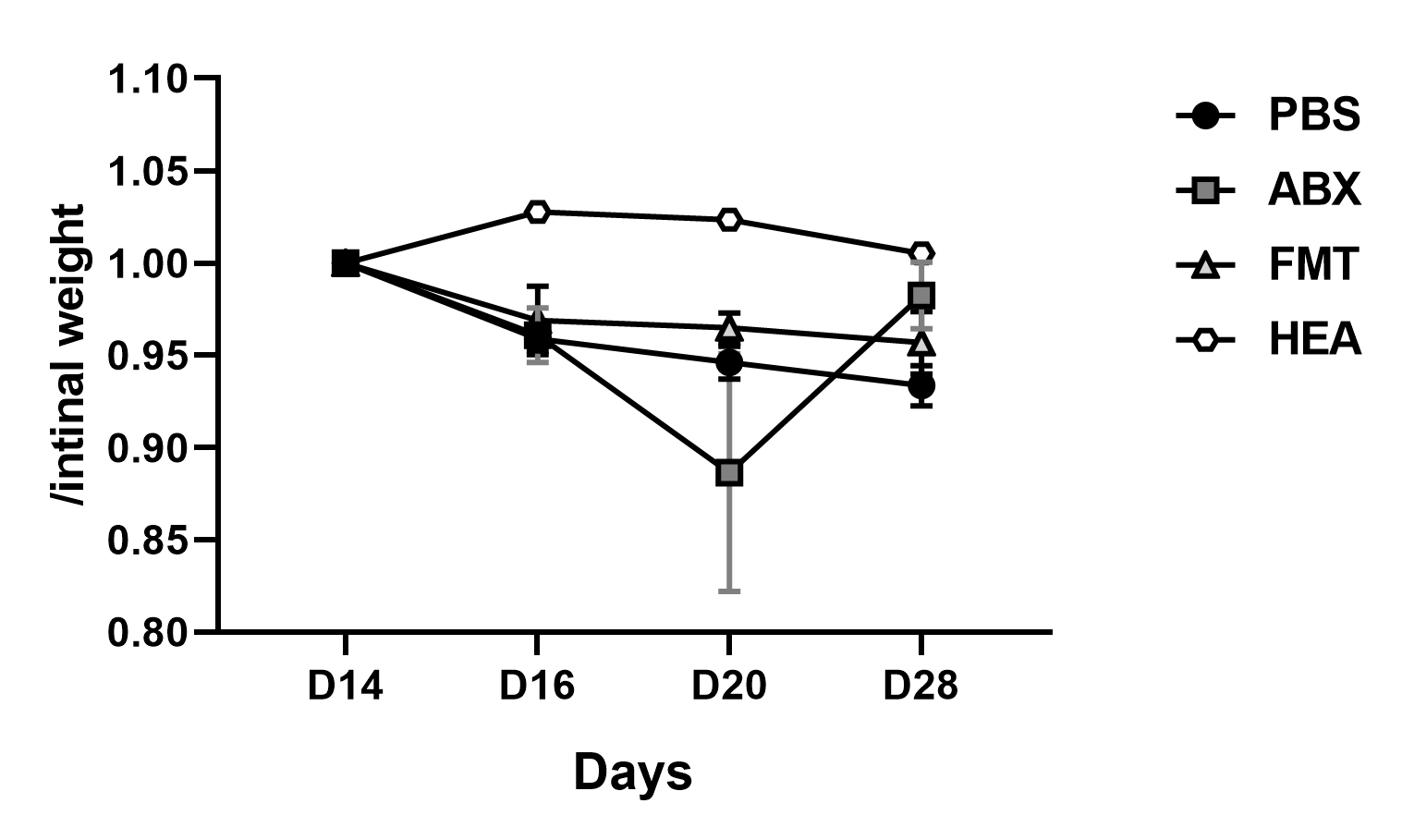

Supplement: Figure S2 — The weights of lambs recorded during the experiment. [file msystems.00953-23-s0002.tif]

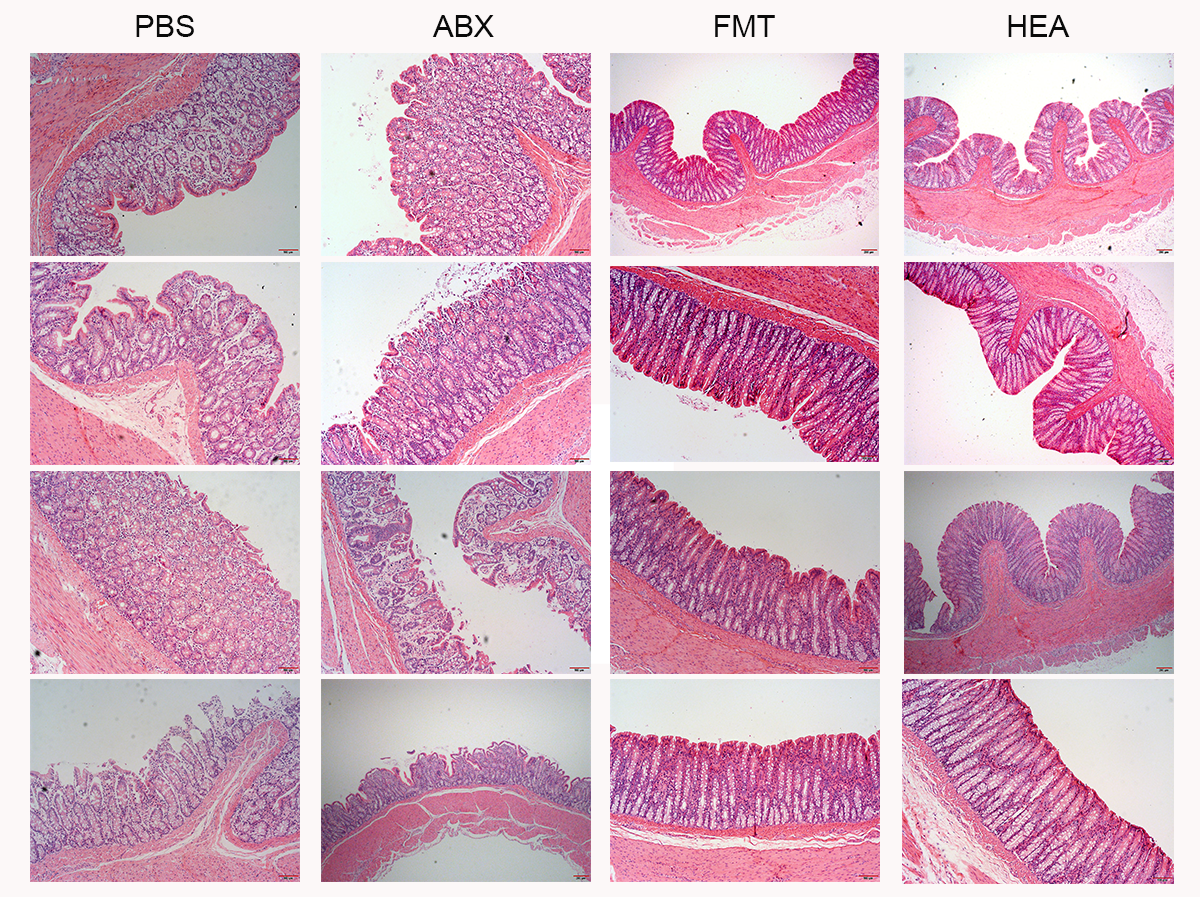

Supplement: Figure S3 — Hematoxylin-eosin staining of colonic tissue in the lambs. [file msystems.00953-23-s0003.tif]

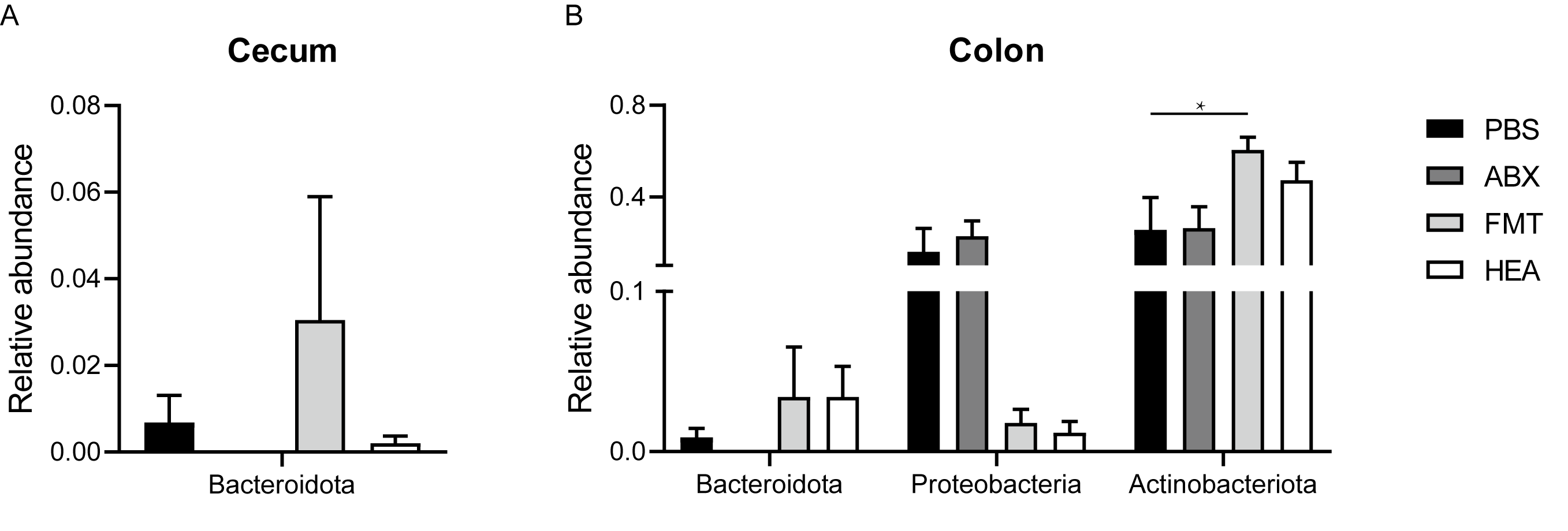

Supplement: Figure S4 — FMT significantly alters the abundance of intestinal bacteria. phylum level abundance of cecum (A) and colon (B) contents. *, P < 0.05. [file msystems.00953-23-s0004.tif]

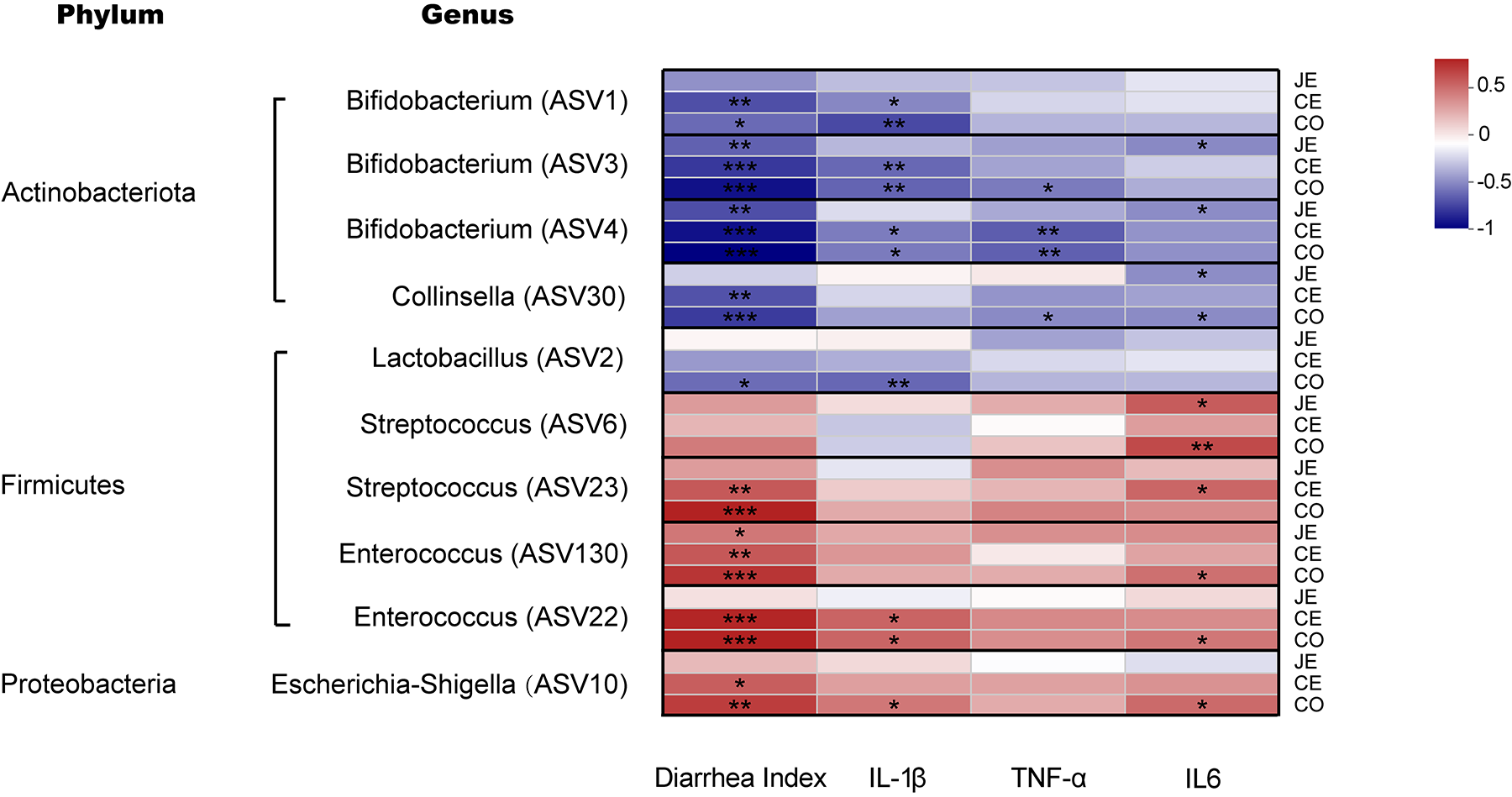

Supplement: Figure S5 — Correlation analysis of intestinal bacteria with diarrhea index and serum inflammatory factor levels in lambs. Red represents a positive correlation, while blue represents a negative correlation. JE, jejunum; CE, cecum; CO, colon. *, P < 0.05; **, P < 0.01; ***, P < 0.001. [file msystems.00953-23-s0005.tif]

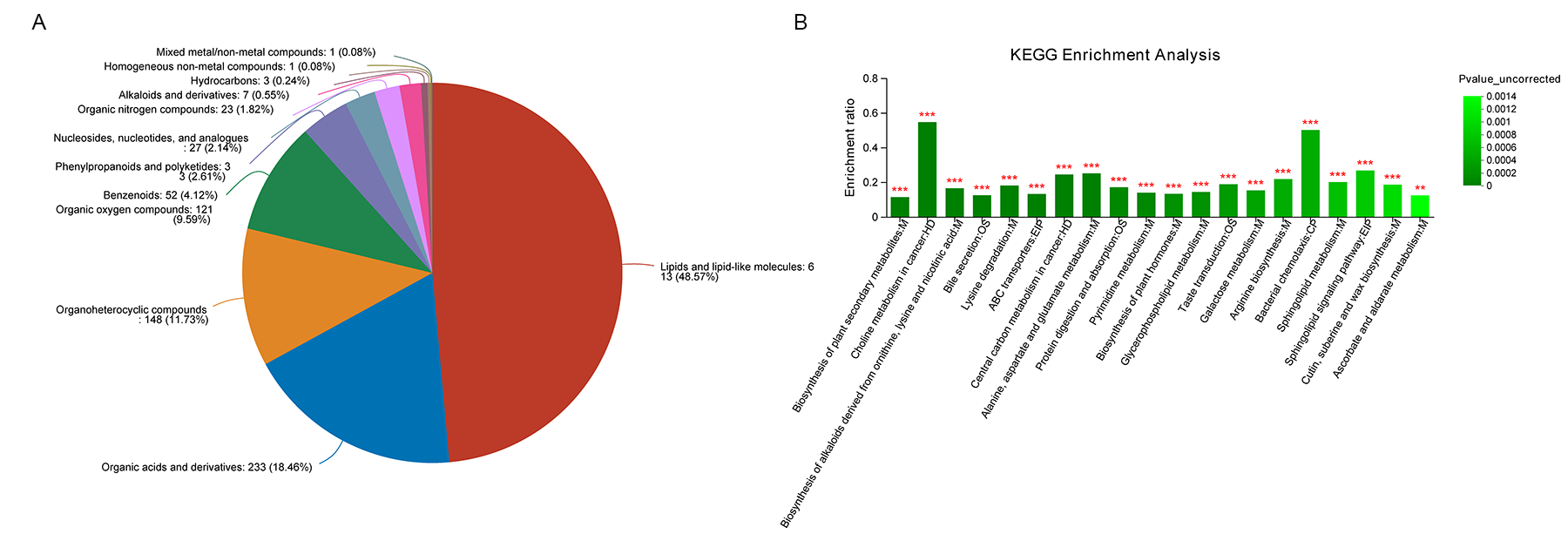

Supplement: Figure S6 — FMT altered the metabolites of the colonic contents of weaned lambs. Taxonomic information and enrichment analysis of metabolites in colonic contents. (A) The detected metabolites were classified as HMDB compounds. (B) The differential metabolites of the four groups were subjected to KEGG pathway enrichment analysis. [file msystems.00953-23-s0006.tif]
